# Molecular mimicry: ecology evolution and applications of doppelgänger peptides

**DOI:** 10.1016/j.tibs.2025.06.011

**Published:** 2025-07-16

**Authors:** Thomas L. Koch, Samuel D. Robinson, Helena Safavi-Hemami

**Affiliations:** 1 Department of Biochemistry, University of Utah, Salt Lake City, UT 84112, USA; 2 Department of Biomedical Sciences, University of Copenhagen, Copenhagen N 2200, Denmark; 3 Institute for Molecular Bioscience, University of Queensland, Brisbane, QLD 4072, Australia; 4 School of Biological Sciences, University of Utah, Salt Lake City, UT 84112, USA; 5 Department of Molecular Pharmaceutics, University of Utah, Salt Lake City, UT 84112, USA

## Abstract

Organisms engage in chemical interactions that drive cooperation, conflict, natural selection, and adaptation. Among these, doppelgänger peptides molecular mimics of the endogenous hormones or neuropeptides of another organism) have evolved in many venomous and poisonous organisms, and some parasites and pathogens. While the discovery of these peptides has been largely anecdotal, a surge in sequence data combined with computational tools suggests they are more prevalent than previously recognized. Beyond their significance in biology, emerging techniques for studying cellular signaling and a renewed interest in peptide-based therapeutics position these molecules as candidates for translational applications. In this review, we explore the role of doppelgänger peptides in chemical ecology, molecular evolution, and medicine, and provide new perspectives to guide future research.

## Chemical interactions in nature

Organisms exist within ecosystems where they interact with members of their own and other species. These interactions form the basis of ecosystems, shaping the survival, reproduction, and evolution of the participants. Generally, such interactions range from mutually beneficial to conflicting, as seen in competition, predation, and parasitism [1]

Conflicting interactions are powerful drivers of natural selection, often giving rise to evolutionary arms races [2]. While many adaptations arising from conflicts can be categorized as morphological, many organisms navigate conflicts via chemical interactions. Chemical compounds that mediate organismal interactions, known as semiochemicals, have critical roles in processes such as mate attraction, predator deterrence, and prey localization [3].

One of the means that organisms use to deceive and manipulate one another in chemical interactions is molecular mimicry. Instead of mimicking morphological traits, molecular **mimics** (see Glossary) show biochemical similarities to molecules of the target organism. By releasing or injecting such molecules, the producer can manipulate critical physiological or behavioral processes of the target organism conferring adaptive advantages.

**Figure 1.**
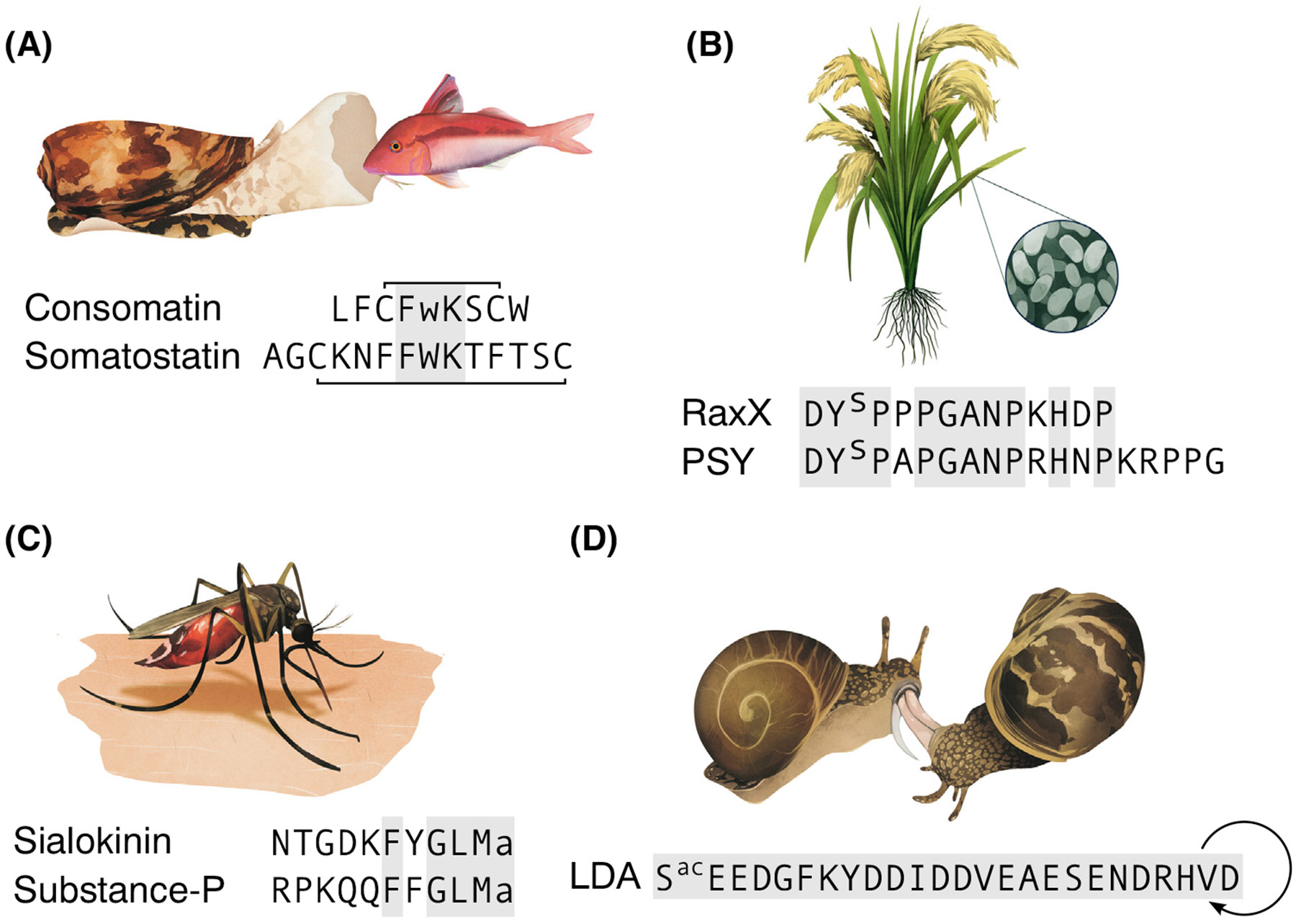
Diverse biotic interactions mediated by doppelgänger peptides. (A) The venomous cone snail *Conus geographus* uses consomatin pG1, a mimic of the peptide hormone somatostatin, to catch fish prey. (B) The pathogenic bacterium *Xanthomonas oryzae* pv. *oryzae* secretes the peptide RaxX, which mimics the phytohormone Plant Peptide Containing Sulfated Tyrosine (PSY) to manipulate its host plant. (C) Mosquitoes inject the peptide sialokinin into the bloodstream of their host as part of their saliva. Sialokinin mimics the vertebrate neuropeptide substance P. (D) Hermaphroditic land snails use love darts during mating to inject the peptide love dart allomone (LDA) and other neuropeptides, which are identical to the endogenous peptides of the snail and influence reproductive processes in the partner. Identical residues in doppelgänger peptides and signaling peptide are highlighted. All peptides shown contain post-translational modifications: a, C-terminal amide; connecting lines, disulfide bonds; S^ac^, actyl-serine; w, D-tryptopha; Y^s^, sulfotyrosine. Artwork by Ilusea Studio.

One example of molecular mimicry is **doppelgänger peptides**, which mimic peptide hormones or neuropeptides in the target organism. While their discovery has been mostly anecdotal to-date, with isolated findings across different systems, technological and conceptual advances are now uncovering their widespread presence. Here, we examine the concept of doppelgänger peptides and their unifying features. We present concrete, illustrative examples to introduce the phenomenon (Figure 1), followed by an exploration of the underlying mechanisms, implications for evolutionary biology, ecology, and pharmacology, and, lastly, a consideration of the future of this field.

## Doppelgänger peptides in nature

### Doppelgänger peptides are prevalent in venomous and poisonous animals

Venoms and poisons consist of bioactive compounds that disrupt physiological and behavioral processes of the target organisms [4]. Many venomous and poisonous lineages have evolved doppelgänger peptides fulfilling these functions alongside other toxins. Numerous examples of doppelgänger peptides have been documented (Table 1), with the greatest diversity in cone snails and anurans (frogs and toads).

Cone snails are predatory gastropods that use toxins, including doppelgänger peptides, to capture and incapacitate prey. An example of doppelgänger peptides in cone snails is venom insulins that mimic the **endogenous** insulin of their sh prey: venom insulins induce hypoglycemia in the prey, depriving the brain of glucose and triggering a comatose state, which facilitates prey capture [5] (Figure 1A). In at least one species, *Conus geographus*, insulin doppelgängers operate alongside other doppelgänger peptides that mimic the hormone **somatostatin**. These so-called ‘consomatins inhibit pancreatic glucagon secretion by activating the somatostatin 2 receptor, preventing the natural counter-response of the sh to low blood glucose and further driving it into an state of hypoglycemia [6].

Many anurans secrete poisons from their skin that contain peptides that help deter predators. The first doppelgänger peptides identified in anuran skin secretions were mimics of the hormones bradykinin and tachykinin [7,8] (Table 1). Similar to their endogenous counterparts, anuran skin bradykinins and tachykinins have vasoactive and myotropic effects, inducing either smooth muscle contraction or relaxation, which may induce pain, vomiting, and diarrhea upon ingestion [9]. Various other doppelgängers have been identified in anuran skin secretions, including those that resemble opioids, gastrin, gastrin-releasing peptide, and neurotensin (Table 1).

### Doppelgänger peptides are utilized in a range of chemical interactions

While the role of several doppelgänger peptides in venomous and poisonous interactions is well documented, their presence extends to other types of chemical interaction. From pathogens and parasites to sexual conflicts, these molecular mimics have evolved to manipulate hosts, evade immune defenses, and influence mating behaviors.

One example is the bacterium *Xanthomonas oryzae*, which infects rice and causes blight. In response to infection, plants initiate an immune response coordinated by various phytohormones [10]. To counteract this, *X. oryzae* produces RaxX, a peptide that mimics the phytohormone Plant Peptide Containing Sulfated Tyrosine (PSY) [11] (Figure 1B). PSY is a regulator of growth and immunity and, by imitating it, RaxX disrupts the natural defense mechanisms of the plant and promotes abnormal growth, which facilitates bacterial colonization [11].

Some pathogenic viruses likewise utilize peptides to evade immune defenses and manipulate host physiology [12,13]. Viruses from the *Iridoviridae* produce viral insulin/insulin-like growth factor (IGF)-like peptides capable of binding to human insulin and IGF-1 receptors [14], lowering blood glucose levels and activating downstream receptor signaling, although their function during infection remains unknown.

Blood-feeding parasites also deploy doppelgänger peptides to disrupt vertebrate hemostasis. The mosquito *Aedes aegypti* injects sialokinin, a mimic of substance P, to activate host neurokinin receptors at the bite site (Figure 1C) [15]. This induces nitric oxide release, promoting blood profusion and counteracting hemostasis. Similarly, ticks of the genus *Ornithodoros* secrete a doppelgänger of the vertebrate peptide adrenomedullin, a potent vasodilator [16].

Some doppelgänger peptides are also implicated in sexual interactions. Since reproduction is heavily regulated by peptides [17,18], manipulative and deceptive peptides have evolved to influence mating behaviors and outcomes. One example comes from hermaphroditic snails, which use ‘love darts to inject bioactive compounds into their mating partner (Figure 1D) [19]. In the snails *Theba pisana* and *Cornu aspersum*, these darts deliver several neuropeptides, including the molluscan orthologs of oxytocin, conopressin, and LDA [20,21]. These peptides have endogenous functions in reproduction, but, by injecting the identical peptides **exogenously**, the snail can obtain control of the reproduction of their mating partner. For instance, LDA contracts the copulatory canal in the injected individual, thereby delaying the digestion of the donated sperm, increasing paternal outcomes [20]. The function of other love dart peptides remains unknown, but their presence suggests a chemical strategy to enhance reproductive success.

## Susceptibility of peptide signaling systems to molecular mimicry

Chemical interactions between organisms involve a range of compounds, from small molecules to lipids, peptides, and proteins, as well as numerous molecular targets. For instance, toxins range from enzymes that degrade tissue to ion channel-blocking peptides that induce paralysis [22]. Among these diverse strategies, why have doppelgänger peptides evolved in so many organisms?

To understand this, one must consider the fundamental role of peptides in intercellular communication across all life forms. While both peptides and small molecules contribute to signaling, peptides represent the most abundant and versatile family of signaling molecules [23]. In humans alone, hundreds of signaling peptides have been identified, with many more likely to be discovered [24]. However, peptide signaling extends beyond animals. In plants [25], fungi [26], and even single-celled organisms [27,28], peptide-based systems have evolved to regulate physiological processes.

In animals and, to a lesser extent, in other domains of life, peptides have key roles in most physiological and behavioral processes, including metabolism, pain perception, immunity, reproduction, learning, development, and growth [29]. Therefore, it is not unexpected that many organisms have evolved mechanisms to hijack peptide signaling systems, gaining control over the physiological processes they regulate.

To do so, peptide receptors are targeted. In animals, most signaling peptides exert their effects by activating G protein-coupled receptors (GPCRs) [30], although other receptor types exist. For example, insulins signal through receptor tyrosine kinases (RTKs) [31], while most plant peptides operate through leucine-rich repeat (LRR) receptor kinases [32]. While it is possible to modulate these receptors with non-doppelgänger peptides (e.g., mambaquearetin-1, which targets the vasopressin V2 receptor but does not mimic the ligand vasopressin [33]), most known cases involve doppelgänger peptides, although this may partly reflect a bias in detection methods.

Peptide receptors activate upon binding of their cognate peptide ligands, rendering them particularly vulnerable to doppelgängers that closely resemble these ligands. Although peptide receptors show specificity for their peptide ligand(s), many retain notable flexibility in recognizing structurally similar ligands, which also form the basis for therapeutic peptide analogs [34,35]. This creates opportunities for exploitation by organisms that deploy doppelgänger peptides.

An additional reason why peptide signaling pathways may be particularly attractive targets is the potential difficulty associated with evolving resistance. Most signaling peptides and their cognate receptors share an ancient coevolutionary history [30]. Resistance would require coordinated escape by both the endogenous peptide and its receptor, essentially moving the system to a new state no longer ‘occupied by the doppelgänger. However, doppelgänger peptides frequently appear as multiple variants (e.g., [36]), collectively occupying the ‘peptide space that the receptor recognizes, further complicating the evolution of resistance. To the best of our knowledge, the only example of resistance comes from the rice *Oryza longistaminata*, which has evolved resistance to the bacterial PSY-mimic, RaxX. In addition to having the cognate PSY receptor, *O. longistaminata* has evolved a decoy receptor, XA21, which binds RaxX with higher af nity to initiate an immune response [11].

## Effects of doppelgänger peptides as extended phenotypes: mimicry beyond the organism

**Figure 2.**
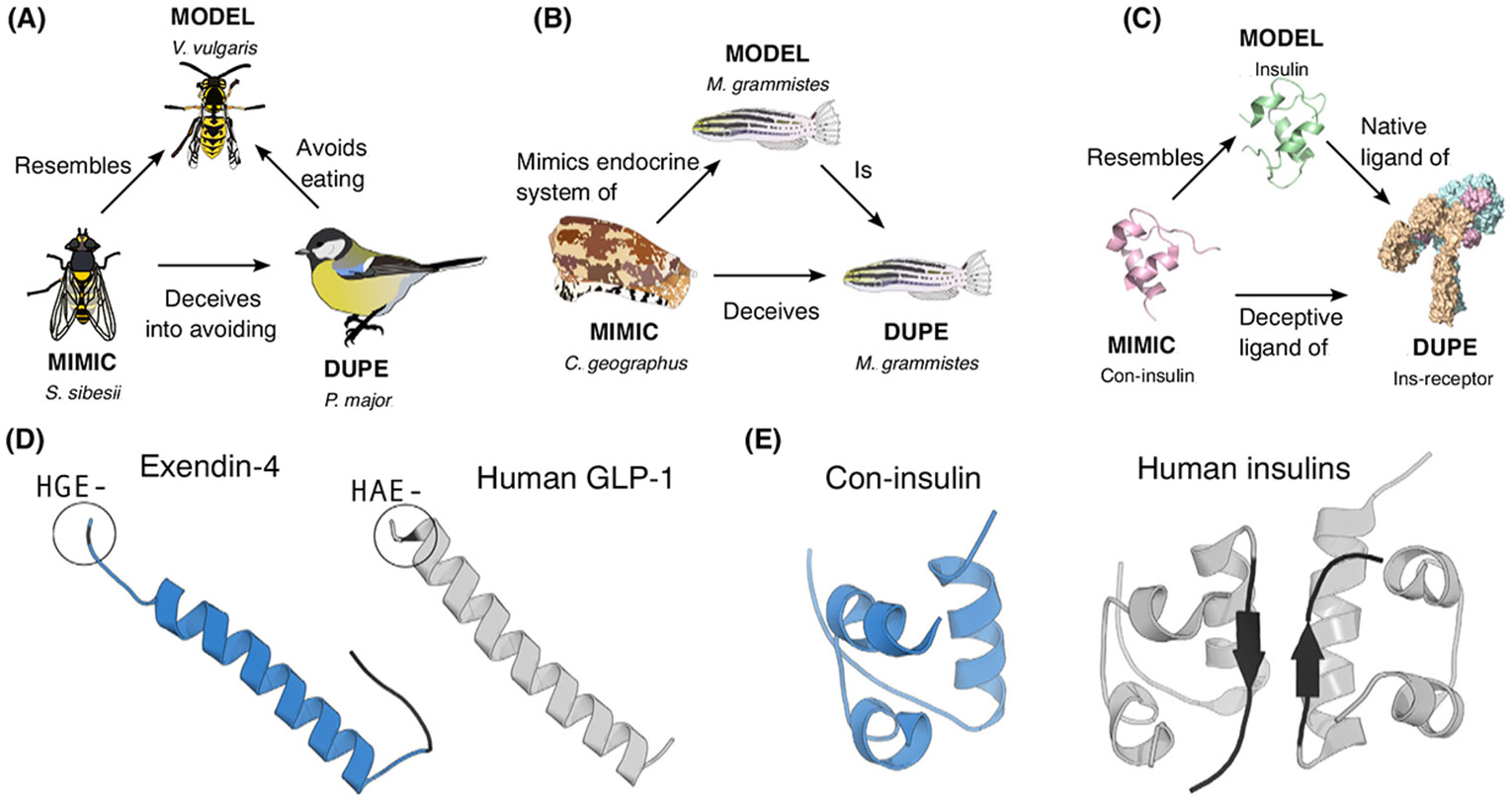
Molecular mimicry at multiple levels. (A) Using Batesian mimicry, the hoverfly *(Syrphus sibesii)* resembles the venomous wasp *(Vespula vulgaris)* deceiving its bird predator (e.g., *Parus major*). Birds learn to avoid wasps due to their sting, and, by mimicking the wasp, the hoverfly deceives the bird into avoidance behavior. (B) Doppelgänger mimicry from an organismal perspective, where the venomous cone snail (*Conus geographus*) produces venom components that mimic components of the endocrine system of its fish prey, deceiving it. (C) Doppelgänger mimicry from a molecular perspective, *C. geographus* produces con-insulin, a doppelgänger peptide that mimics the endogenous insulin of the prey, activating insulin receptors in the latter. (D) Exendin-4 mimics glucagon peptide-like 1 (GLP-1), but has several unique features (black residues), including an N-terminal HGE motif that reduces enzyme-mediated peptide degradation, and a C-terminal extension. (E) Con-insulin mimics endogenous insulins but lack the C-terminal extension of the B-chain (black residues), which is responsible for dimer formation in human insulin.

Mimicry systems can be described as a mimic that resembles a **model** to deceive a **dupe** [37]. For instance, some hoverflies resemble stinging wasps to reduce predation (Figure 2A). How do doppelgänger peptides t into the mimicry framework?

The deception of doppelgänger peptides can be analyzed from both organismal and molecular perspectives. From an organismal perspective, the doppelgänger peptide-producing organism (mimic) mimics its target organism, which acts as both the model and the dupe (Figure 2B). For example, by producing sh-like insulin, the cone snail imitates a component of the endocrine system of the sh it aims to deceive [5].

From a molecular perspective, the doppelgänger peptide (mimic) mimics a signaling peptide (model) to deceive a receptor (dupe) (Figure 2C). The receptor is unable to distinguish between its natural ligand and the mimic, which sends a deceptive signal. For example, con-insulin mimics sh insulin to activate insulin receptors in the sh [5]. Importantly, the resemblance is from the ‘point-of-view of the receptor. In some cases, a mimic may appear dissimilar at the sequence or structural level but can still effectively engage the receptor. For example, some large plant cyclotides can activate the oxytocin receptor, even though only a small, embedded motif resembles oxytocin [38]. This limited similarity may be suf cient for receptor binding, but is easily overlooked.

Mimicry can be classified into different types, such as **Batesian, Müllerian**, and **Gilbertian**, which also transfers to doppelgänger peptides [37]. For example, the bull ant, *Myrmecia gulosa*, uses the toxin MIITX_2_-Mg1a, a mimic of epidermal growth factor (EGF), which binds the vertebrate receptor and delivers a strong, long-lasting painful stimulus. EGF mimicry by the bull ant can be regarded as Gilbertian mimicry because the dupe is repulsed by the painful stimulus of the EGF-like toxin [39]. As such, most defensive cases of doppelgänger peptides can be thought of as Gilbertian. However, aggressive uses of doppelgänger peptides, such as the insulin mimicry of cone snails, align with Batesian-Wallacian aggressive mimicry, where the predator disguises as the model to incapacitate it [5].

The classification of mimicry type is dependent on the biological function of the peptide in the interaction. Doppelgänger peptides are fundamentally an ecological concept defined by the interaction they mediate and should also be examined in this context, although this can be complicated by the dynamic nature of most organismal interactions. Broadly speaking, doppelgänger peptides transmit deceptive signals that prompt the target organism to act in the interest of the transmitter. In this way, beyond mimicry, the physiological or behavioral effects that doppelgänger peptides exert can be understood as extended phenotypes (traits that extend beyond the organism itself [40]) of the genes that encode them.

## Unifying molecular and biochemical characteristics of doppelgänger peptides

Doppelgänger peptides often closely resemble the endogenous peptides they mimic, and variations in their sequence can often be linked to distinct functional differences. Both endogenous peptides and doppelgänger peptides originate from larger **precursor proteins** [41]. Many doppelgänger toxins are derived from peptide hormone precursors, inheriting their structural framework (Box 1). These precursors include an N-terminal signal sequence that directs the nascent peptide chain to the secretory pathway. Maturation involves cleavage of the active peptide from the propeptide/spacer region and the addition of post-translational modifications that can enhance potency and stability.

**Figure 3.**
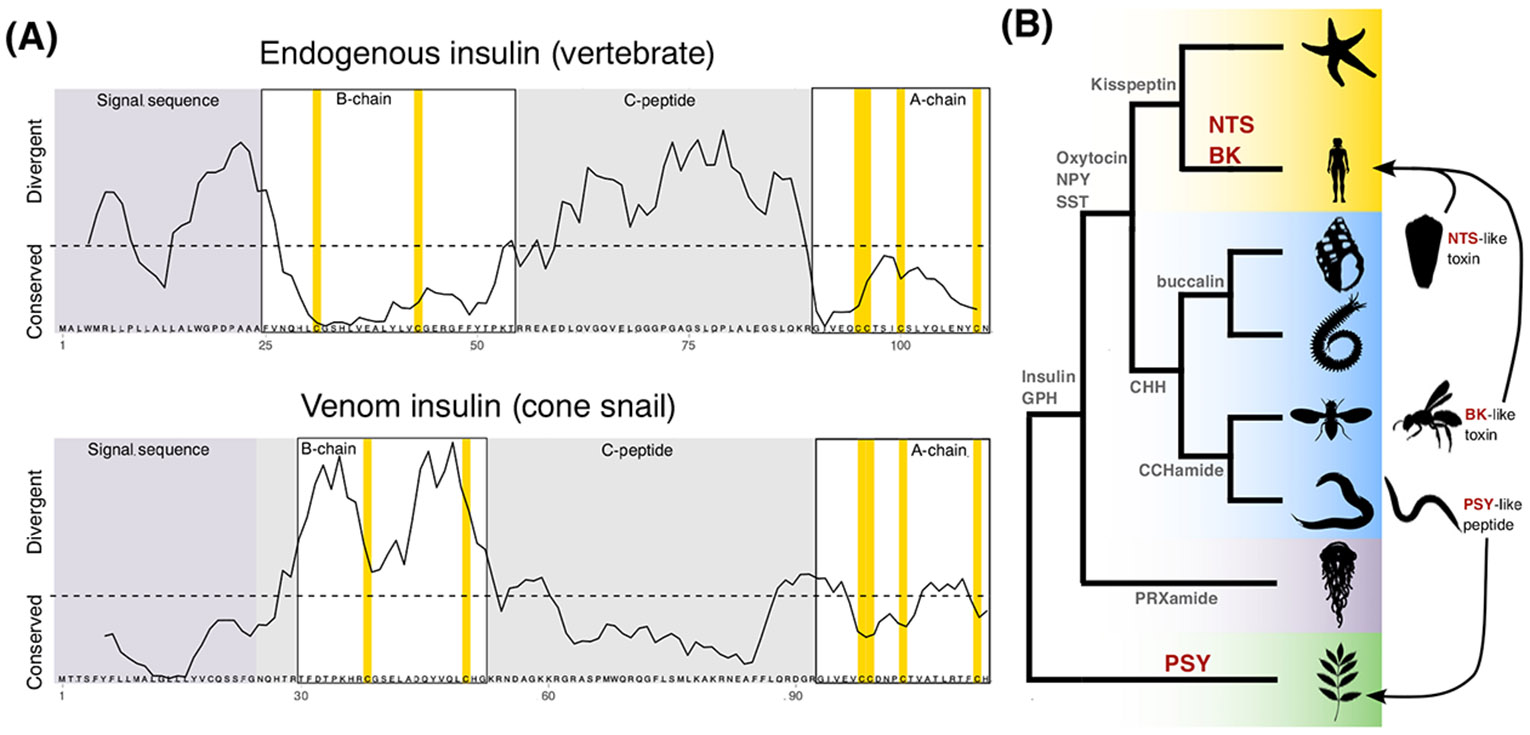
Evolutionary patterns of doppelgänger peptides. (A) Endogenous peptides and doppelgänger peptides have contrasting patterns of molecular evolution. The curve shows the moving average of the conservation score (rate4site as used in [36]) for a multiple sequence alignment of vertebrate insulin and cone snail doppelgänger insulin. While endogenous peptides are usually highly conserved in the region harboring the active peptide (example shown: the B- and A-chains of vertebrate insulins), doppelgänger peptides are highly divergent in these regions and conserved in the regions that harbor the signal sequence and spacer regions (in gray, example shown: C-peptide of venom insulins). (B) Schematic phylogenetic tree of eukaryotes illustrates the lineage-specific emergence of selected signaling peptides [e.g., neurotensin (NTS) and bradykinin (BK) in chordates, and Plant Peptide Containing Sulfated Tyrosine (PSY) in plants]. The discovery of doppelgänger peptides that mimic lineage-specific peptides in unrelated organisms suggests ecological interactions between the producer and the target species, providing insights into ecological interactions.

The evolutionary conservation patterns of doppelgänger peptide precursors contrast with those of their endogenous counterparts (Figure 3A) [36,42]. In endogenous peptides, the biologically active region is usually highly conserved. Coevolution with cognate receptors imposes strong evolutionary constraints, maintaining sequence stability. The remainder of the precursor serves primarily as a structural framework and is more variable. In several documented cases, the precursors encoding doppelgänger peptides exhibit the opposite pattern [36,42,43]. The region encoding the active peptide evolves rapidly compared with other regions of the precursor, creating a recognizable pattern that could be exploited to identify doppelgänger peptides. While this pattern has been observed in some cases, it is not known whether it is a universal feature or one that depends on the ecological context.

Interestingly, doppelgänger peptides rarely achieve perfect mimicry, even when identical sequences are theoretically possible. In classical mimicry theory, it has been assumed that selection favors increasingly accurate resemblance to the model [44], yet doppelgänger peptides often exhibit ‘imperfect mimicry . Several factors likely contribute to this phenomenon: (i) receptor promiscuity: many peptide receptors exhibit flexibility, allowing them to be activated by structurally similar peptides. This reduces the requirement for precise mimicry, because imperfect resemblance can still result in sufficient biological activity: (ii) target variability: doppelgänger peptides may be targeted at multiple different species, each with slightly different endogenous peptides. This variation may drive the evolution of peptides that are less pharmacologically selective: (iii) adaptive constraints: in some cases, mimics may face trade-offs between perfect mimicry and other functional constraints. For example, if a doppelgänger peptide retains its endogenous functions in the producer organism, this moonlighting function could prevent perfect mimicry of the endogenous peptide of the target organism. When doppelgänger peptides arise *de novo* or from gene duplication, they may avoid such conflicting evolutionary pressures, in which case imperfect mimicry may reflect adaptive advantages: and (iv) the selective advantages of imperfect mimicry can provide doppelgänger peptides with novel functional properties. Despite sharing target receptors, doppelgänger and endogenous peptides have different biological functions.

Endogenous peptides function in intercellular communication, often requiring precise spatial and temporal regulation. Close proximity between peptide release and reception and short half-lives allow for tight spatiotemporal control of signaling [23,45]. By contrast, doppelgänger peptides are **exochemicals**. Produced by a different organism, they must remain stable long enough to reach their target receptors, necessitating increased resistance to degradation. In addition, while endogenous peptides may activate multiple receptor subtypes, because doppelgänger peptides are typically not delivered near their specific target, they may, in some cases, evolve selectivity for specific receptor subtypes to achieve maximum physiological outcomes [6,46].

Thus, while their mimicry may be imperfect, doppelgänger peptides are functionally adapted to the ecological and physiological demands of their role.

## Doppelgänger peptides overcome longstanding challenges in drug design

Given their roles in regulating diverse physiological processes, endogenous signaling peptides are valuable as drug leads for a range of diseases [47]. Several landmark peptide-based drugs exemplify their therapeutic potential, such as insulin for diabetes [48], oxytocin for labor induction [49], and glucagon-like peptide 1 (GLP-1) and other incretins for the management of type 2 diabetes mellitus and obesity [50].

Despite these advances, significant challenges remain in developing signaling peptide-based therapeutics. Native signaling peptides have limitations restricting their broader therapeutic utility, arising from the biological constraints associated with their functional roles. While advantageous in the native context, these properties, such as a short half-life, low bioavailability, and receptor promiscuity, limit their utility as drugs.

Peptide-based drugs must be selective, able to navigate extracellular environments, resist enzymatic degradation, and achieve sufficient tissue distribution. Doppelgänger peptides offer an elegant solution to address these challenges. These peptides are delivered via injection, ingestion, or absorption and evolve under selective pressures for high stability, potency, bioavailability, and often specificity. For example, exendin-4 is a doppelgänger toxin found in the salivary gland of the Gila monster (*Heloderma suspectum*), which mimics the peptide hormone GLP-1 and potently activates the human GLP-1 receptor [51]. However, unlike GLP-1, exendin-4 has several modifications that increase its potency and half-life, including a C-terminal extension and the absence of a proteolytic cleavage site found in GLP-1 (Figure 2D) [51,52]. These features led to the approval of exendin-4 as the rst-in-class GLP-1 analog for the treatment of diabetes [53].

Decades after its discovery, the biological function of exendin-4 in the Gila monster is still not fully understood. Although the salivary glands of the Gila monster produce a range of toxins that are used for predation and defense, exendin-4 may not only function as a toxin, but also have a role in internal metabolic processes in the animal. This is supported by the presence of high levels of exendin-4 in the blood of the Gila monster following a meal [54]. In this scenario, exendin-4 is a doppelgänger peptide that mimics the endogenous gut GLP-1 of the Gila monster, but, because of its oral secretion, has evolved to be more stable than its gut counterpart.

Given their drug-like properties, doppelgänger peptides can either directly serve as drug leads or inform the design of new peptide-based analogs. The latter process can be likened to an evolutionary-informed mutational scanning strategy used in protein engineering. Here, advantageous traits from doppelgängers are transferred to native human peptides. By identifying the evolutionary ‘peptide space that shapes these traits, one can pinpoint key amino acid residues or structural motifs to optimize function and graft these onto native peptides to create analogs that minimize the risk of immunogenicity, a potential concern with nonhuman-derived peptides. An example of this approach is the design of monomeric, fast-acting analogs of human insulin based on venom insulins from sh-hunting cone snails that lack the classical B-chain C terminus, which causes dimerization of endogenous insulin (Figure 2E) [55,56] (and see above).

## Inferring biotic interactions through doppelgänger peptides

Signaling peptides used in various organisms have different evolutionary histories. Many are highly conserved across the animal kingdom, with some dating back to prebilaterian linages, such as insulin-like peptides and glycoprotein hormones (Figure 3B) [57]. However, other peptides have evolved more recently. For instance, neurotensin is a chordate-specific neuropeptide, and crustacean hyperglycemic hormone (CHH) is only found in protostomes [30,58]. As a result, each organism has a specific complement of peptides that regulate its internal physiology. Consequently, identifying a doppelgänger peptide that mimics a lineage-specific peptide can reveal that the producer organism interacts with an organism from that lineage, thereby providing ecological information (Figure 3B). For instance, identifying a neurotensin-mimicking doppelgänger peptide in a venomous species implies that it targets chordates, since neurotensin is exclusive to this lineage. This principle is exemplified by Contulakin-G, a neurotensin mimic found in the sh-hunting *C. geographus* [59]. Similarly, doppelgänger peptides of the plant phytohormone PSY have only been found in nematodes that parasitize plants [60]. Thus, if a PSY-mimicking peptide were to be discovered in a nematode species with unknown biology, one could reasonably infer that it is also a plant parasite.

Even in cases where peptide families are shared across multiple phyla, a combination of sequence divergence between endogenous peptides and similarity of doppelgängers can help to pinpoint specific interactions. Both insulin and somatostatin are found across multiple phyla, but cone snail doppelgängers closely mimic those of their respective prey [36,61]. In some instances, mimicry specifically extends to the species level, as seen in the EGF-mimicking toxin of the Australian giant red bull ant, which most closely resembles the EGF of its main predator, the echidna (*Tachyglossus aculeatus*) [39]. In addition, shifts in prey preferences over the lifetime of an organism can drive corresponding changes in doppelgänger peptide production, representing a form of developmental mimicry. For instance, juvenile magician cone snails, *Conus magus*, which prey on annelids, produce annelid-like somatostatin peptides, whereas adults, which shift to a sh diet, produce sh-like somatostatin peptides [62]. Finally, the mimicry can extend to tissue/circuit-specific isoforms of peptides. For example, consomatins from *C. geographus* closely resemble one isoform of somatostatin, SS4, found in the Brockman bodies of sh, an organ analogous to the pancreas [6].

These examples illustrate how doppelgänger peptides can, in principle, reveal biotic interactions in systems that remain ecologically uncharacterized. As large-scale sequencing efforts continue to outpace ecological data collection, such molecular insights may become increasingly valuable, offering a way to infer novel ecological relationships.

## Discovery of novel signaling peptides through doppelgänger peptides

Since the rst human signaling peptides were discovered during the 1920s and 1930s, hundreds have been characterized. However, the presence of numerous orphan peptide receptors and an increasing number of putative small open reading frame-encoded peptides suggest that many human peptides remain undiscovered. The challenge of identifying these peptides both experimentally and computationally stems from their often low and temporally restricted expression, as well as their molecular characteristics [24,63,64] (Box 2).

Doppelgänger peptides offer a promising strategy to partially address this challenge. While most doppelgänger peptides have been identified based on their resemblance to known signaling peptides, this process can also be reversed, leading to the discovery of previously unrecognized peptides. For example, the doppelgänger peptide bombesin, which can induce gastrin release and exert potent activity in the mammalian nervous system, was rst isolated from the skin secretions of the toad *Bombina bombina* [65]. Initially, bombesin was thought to be a signaling peptide and this discovery spurred the search for a similar human peptide, which led to the identification of the peptides hormones gastrin-releasing peptide and neuromedin-B [66]. Only later was it realized that bombesin was a doppelgänger of these peptides, which may have evolved to induce vomiting if ingested [65]. Similarly, somatostatin mimics from cone snail venom facilitated the discovery of novel somatostatin-like peptides in protostomes [36], and the cnidarian toxin Shk-like2 guided the identification of a cnidarian neuropeptide [67]. The endogenous functions of these newly discovered neuropeptides remain to be elucidated.

This ‘doppelgänger-first approach is particularly attractive because doppelgänger discovery is often easier than identifying their endogenous counterparts. Doppelgänger peptides tend to be highly expressed in specialized glands, simplifying their sequencing and characterization. Furthermore, doppelgänger peptides also exhibit distinct molecular characteristics that facilitate their detection, such as different rates of evolution across the precursor (Figure 3A) and their birth by gene duplication (Box 1). Given these advantages, doppelgängers can serve as elegant tools for uncovering novel signaling peptides. This approach recently led to the identification of a previously unrecognized lophotrochozoan CHH-like peptide and several additional neuropeptide candidates [42], suggesting that this strategy can be broadly applied across diverse doppelgänger and neuropeptide signaling systems.

## Concluding remarks

Deception is widespread in nature, with organisms evolving diverse mechanisms to manipulate the behavior and physiology of others [68]. Many of these interactions are mediated by semiochemicals that convey information between species or individuals. From pheromones that influence mating behaviors [69] to allelochemicals that inhibit competitor growth [70], chemical deception has a crucial role in ecology. Among these strategies, molecular mimicry of peptides represents an intriguing form of chemical manipulation, where doppelgänger peptides mimic endogenous signals to control the target organism.

Since the rst discoveries of doppelgänger peptides expressed in unexpected tissues, such as venom and skin [65,71], reports of such peptides have gradually increased, although often under different labels. However, these discoveries have largely been incidental, emerging within broader peptide annotation efforts. Despite their functional parallels with toxins, doppelgänger peptides exhibit unique characteristics that set them apart from conventional toxins. A major challenge in understanding their role has been the absence of a unified conceptual framework and limited cross-disciplinary awareness. We propose that the diversity and prevalence of doppelgänger peptides are significantly underestimated and advocate for their investigation across a wider range of biological systems, such as the microbiome and cancer cells (see Outstanding questions).

Moreover, relatively few studies have experimentally determined the effects of doppelgänger peptides on the target organism. Establishing these connections is essential to understand their biological significance. Fortunately, recent advances in sequencing technologies, bioinformatics, and functional assays now enable the systematic identification and characterization of these peptides [42]. As new tools continue to emerge, we anticipate rapid expansion of this field, shedding light on the extensive role of peptide-mediated manipulations in ecology and evolution.

## Figures and Tables

**Table 1.** Examples of doppelgängers used in diverse systems

Endogenous hormone or neuropeptide of target	Doppelgänger peptide	Producer organism lineage (species)	Refs
Venoms and poisons			
Angiotensin	Crinia-angiotensin	Anura (*Crinia georgiana*)	Skin secretions [81]
Bradykinin	Bradykinin, kallidin	Anura (*Rana esculenta, Rana temporaria, Phyllomedusa sauvagi*)	Skin secretions [7,8]
	Bradykinin	Hymenoptera (*Vespula vulgaris, Neoponera goeldii*)	Venom [82,83]
Corticotropin releasing factor	Sauvagine	Anura (*P. sauvagi*)	Skin secretions [84,85]
Crustacean hyperglycemic hormone (CHH)	CHH doppelgänger	Conoidea (*Conus textile, Conus marmoreus, Conus furvus*)	Venom [42]
Endomorphin	Dermorphin	Anura (*P. sauvagi*)	Skin secretions [86,87]
Enkephalin	Deltorphin	Anura (*P. sauvagi*)	Skin secretions [87,88]
Epidermal growth factor (EGF)	MIITX_2_-Mg1a	Hymenoptera (*Myrmecia gulosa*)	Venom [39]
Gastrin-releasing peptide (GRP), Neuromedin-B, Neuromedin C	Bombesin (alytesin, litorin, phyllolitorin, ranatensin)	Anura (*Bombina bombina, Alytes obstetricans*)	Skin secretions [65,89]
Glucagon peptide-like 1 (GLP-1)	Exendins	Helodermatidae (*Heloderma suspectum*)	Salivary gland/venom [90]
Insulin	Con-insulin	Conoidea (*Conus geographus*)	Venom [5,61]
Neurotensin	Xenopsin	Anura (*Xenopus laevis*)	Skin secretions [91,92]
	Contulakin-G	Conoidea *C. geographus)*	Venom [59]
Somatostatin and somatostatin-related peptides	Consomatin	Conoidea (*Conus rolani)*	Venom [36,93]
Tachykinin, substance P, substance K, neuromedin K	OctTK-I, OctTK-II	Octopoda (*Octopus vulgaris*)	Venom [74,94]
Parasites			
Adrenomedullin	Tick adrenomedullin	*Icodida* (*Ornithodoros moubata*)	Saliva secretions [16]
Inflorescence deficient in abscission (IDA)	IDA	Nematoda (*Meloidogyne incognita*)	Transcriptome [95]
Plant peptides containing sulfated tyrosine (PSYs)	MigPSY	Nematoda (*Meloidogyne)*	Genome [60]
Tachykinin, substance P, substance K, neuromedin K	Sialokinin I, Sialokinin II	Culicidae (*Aedes aegypti*)	Parasitic secretions [96]
Pathogens			
Endothelin	Viral endothelins	Viruses (Deerpox virus)	Genome [14]
EGF-1/Transforming growth factor (TGF)	Poxvirus growth factors	Viruses (Chordopoxvirinae)	Genome [97-100]
Insulin/Insulin-like growth factor (IGF)	Viral insulin-like peptides (VIPs)	Viruses (Iridoviridae)	Genome [14,101]
PSYs	RaxX	Bacteria (*Xanthomonas oryzae)*	Secretions [11]
Phytosulphokines (PSKs)	PSK-like	Bacteria (*Pseudomonadota)*	Genome [102]
Sexual interactions			
Buccalin-derived love dart allomone (LDA)	LDA	Stymmatophora (*Cornu aspersum)*	Love dart secretions [20]
GLP-1	eGLP-1, pGLP-1	Monotremata (*Ornithorhynchus anatinus, Tachyglossus aculeatus)*	Venom gland [73]

## Highlights

Chemical deception and manipulation are key strategies in biological interactions, often mediated by molecular mimicry.

Doppelgänger peptides, mimics of neuropeptides and peptide hormones, offer selective advantages by altering the physiology and behavior of target organisms.

Although several doppelgänger peptides have been described, their discovery remains challenging. Here, we emphasize unifying characteristics of doppelgänger peptides across diverse biological systems and highlight their significance in biomedicine, ecology, and molecular evolution.

## Box 1. Molecular origins of doppelgänger peptides

Doppelgänger peptides arise primarily through two mechanisms: (i) **recruitment** from an existing peptide family that encodes the peptide they mimic (Figure IA-C) or (ii) **de *novo* gene** birth (Figure ID). Recruitment appears to be the most common mechanism, although this may be skewed by the challenges associated with identifying *de novo* doppelgänger peptides (see Box 2).

Doppelgänger peptides that evolve from a recruitment event can do so through several mechanisms [72]. Most appear to evolve from gene duplication in combination with neofunctionalization and expression in exocrine glands (Figure IB). The order of these events is flexible, and several models have been presented to describe them [72]. Doppelgänger peptides can also emerge from **exon shuffling** involving the combination of exons encoding endogenous signaling peptide genes and other secreted genes, such as those encoding toxins, to generate new chimeric genes (Figure IA) [42]. Another mechanism of recruitment is horizontal gene transfer, as seen in *Ornithodoros* ticks, which acquired adrenomedullin-encoding genes from their vertebrate hosts to facilitate blood feeding [16] (Figure IC). Finally, some doppelgänger peptides, such as glucagon-like peptide 1 (GLP-1) expressed in the venom gland of monotremes, are encoded by the same gene as the endogenous gut-derived peptide and, therefore, act as **moonlighting proteins**/peptides with dual roles [73].

In the case of *de novo* origin, random mutations during periods of neutral selection may result in a ‘smart trick producing a sequence capable of partially activating a signaling peptide receptor (Figure ID). This is not an unlikely event because many peptide receptors recognize relatively short motifs. If this sequence confers an advantage, natural selection can x these mutations, leading to the emergence of a functional doppelgänger peptide. Examples of *de novo* birth include Contulakin-G [59] and octopus tachykinins [74]. The likelihood of a *de novo* doppelgänger peptide arising is inversely proportional to peptide length, making it more likely for a *de novo* peptide to mimic smaller peptides such as Leu-enkephalin (four amino acids) or neurotensin (13 amino acids) compared with larger peptides, such as GLP-1 (30 amino acids) or insulin (51 amino acids).

## Box 2. Overcoming challenges in identifying doppelgänger peptides

Detecting doppelgänger peptides remains a significant bioinformatical challenge, particularly due to their short sequences, rapid evolution, and potential *de novo* origins. Traditional sequence-based approaches struggle to identify these peptides because they are encoded within larger precursor molecules, with the biologically active region spanning only a few conserved residues [57]. Many peptide precursors further fall into the ‘twilight zone of protein similarity [75], where sequence similarity is too weak for methods, such as BLAST, to reliably detect relationships. This challenge is even greater for those doppelgänger peptides that may have originated *de novo* or through rapid sequence divergence.

To address these limitations, more sensitive similarity detection methods are needed. Profile Hidden Markov Models (pHMMs) and PSI-BLAST extend detection capabilities but often remain insufficient for identifying more divergent doppelgänger peptides. Alternative methods, such as fuzzy regular expressions, which incorporate evolutionary information and flexible Levenshtein distances, can increase sensitivity but risk increasing false-positive [6] rates. Lastly, expanding taxonomic sampling can also increase similarity detection. Given that direct sequence similarity erodes over evolutionary time, incorporating intermediate sequences can help bridge gaps between distantly related homologs through transitive relationships [76].

Beyond sequence-based approaches, structure-based methods have emerged as tools for identifying peptide homologs and analogs. Protein tertiary structures evolve more slowly than do primary sequences, meaning that structural similarities are often present even when sequence similarities are undetectable. Tools, such as AlphaFold2 [77] and FoldSeek [78], enable structural similarity searches that can lead to the discovery of novel signaling peptides and their doppelgänger counterparts where sequence-based methods fail. For example, this approach was instrumental in uncovering a previously unrecognized molluscan CHH-like doppelgänger peptide and its endogenous counterpart in lophotrochozoans [42].

Future advances in representation learning are likely to further improve our ability to detect distant similarities between signaling peptides and doppelgänger peptides, even when these similarities are undetectable at the sequence and structural level. As representation learning continues to advance [79], we anticipate improvements in detecting subtle relationships between signaling peptides and doppelgänger peptides, where more traditional similarity-based searches fail.

Other significant conceptual hurdles in the discovery of doppelgänger peptides have been the assumption of orthology and insufficient consideration of convergent evolution. These issues have been particularly pronounced in the study of anuran peptides, where several doppelgängers identified from skin secretions were originally mistaken as orthologs of endogenous vertebrate peptides, rather than recognizing them as convergently evolved doppelgängers adapted to target different organisms [80].

## Outstanding questions

How prevalent are doppelgänger peptides across different taxonomic lineages, and what factors influence their distribution?

Which evolutionary pressures and molecular mechanisms drive the emergence and maintenance of doppelgänger peptides?

How can novel computational and experimental approaches accelerate the discovery and functional characterization of doppelgänger peptides?

What roles do doppelgänger peptides have in mediating sexual conflict and reproductive strategies?

How frequently do organisms evolve resistance to doppelgänger peptides, and what counter-adaptations arise in response?

Can the presence of doppelgänger peptides be used to predict previously unknown biotic interactions?

To what extent do doppelgänger peptides hold therapeutic potential in medicine and biotechnology?

Are there specific physiological or signaling systems that are more commonly targeted by doppelgänger peptides?

How do different types of ecological and symbiotic interaction shape the evolution and function of doppelgänger peptides?

What selective advantages do doppelgänger peptides confer to the organisms that produce them, and how do these benefits vary across contexts?

## Glossary

Batesian mimicry: form of defensive mimicry where a harmless organism gains protection by resembling a dangerous or unpalatable organism.
*de novo* gene: novel gene that arises from a previously non-genic region of the genome, rather than through duplication or modification of existing genes.
Doppelgänger peptide: molecular mimic of a neuropeptide or peptide hormone of the target organism.
Dupe: organism or entity that is deceived by the mimic, responding as though it has encountered the model.
Endogenous: functioning in the body of the producing organism.
Exochemical: chemical compound working outside the body of the producing organism.
Exogenous: functioning outside the body of the producing organism.
Exon shuffling: genetic mechanism that can generate new genes by recombining two or more exons from different genes, creating novel combinations with potentially new functions.
Gilbertian mimicry: form of defensive mimicry where a prey organism gains protection by mimicking one or more features of its predator.
Mimic: organism or entity that imitates the model to deceive the dupe for an adaptive advantage.
Model: in a mimicry system, the entity that is being imitated by the mimic.
Moonlighting protein: phenomenon where a single protein carries out multiple distinct functions, often in different cellular contexts or conditions.
Müllerian mimicry: form of defensive mimicry where two or more unpalatable or harmful organisms gain protection by resembling one another.
Precursor protein: full-length, inactive protein that is processed through enzymatic cleavage and post-translational modifications to generate a biologically active protein or peptide.
Recruitment: process by which molecules with an original physiological function are co-opted for another use, such as toxins in venom or poison systems.
Somatostatin: peptide hormone that regulates a range of effects in the neuroendocrine system, including insulin and glucagon release from the pancreas in humans.
